# Sociodemographic and Other Characteristics Associated With Behavioural Risk Factors of HIV Infection Among Male Mountain-Climbing Porters in Kilimanjaro Region, Tanzania

**DOI:** 10.24248/EAHRJ-D-17-00259

**Published:** 2017-07-01

**Authors:** Jenipher E Lyamuya, Bernard Njau, Damian J Damian, Tara B Mtuy

**Affiliations:** a African Medical and Research Foundation, Moshi, Tanzania; b Kilimanjaro Christian Medical University College, Moshi, Tanzania; c London School of Hygiene & Tropical Medicine, London, UK

## Abstract

**Background:**

Alcohol consumption, marijuana use, unprotected sex, and multiple concurrent sexual partnerships are prevalent among youth globally. These factors are regarded as important behavioural risk factors for HIV infection. The aim of this study was to assess the sociodemographic and other characteristics associated with behavioural risk factors of HIV infection among male mountain-climbing porters working on Mount Kilimanjaro in Tanzania.

**Methods:**

This cross-sectional study enrolled a representative sample of 384 male mountain-climbing porters from 7 tour companies in the Kilimanjaro region using a multi-stage sampling technique. Local interviewers completed a structured questionnaire with porters in the local language, Kiswahili. The questionnaire covered demographics, alcohol and marijuana use, sexual history, sexual partners, and condom use. In-person interviews were completed between April and May 2013. Univariate and bivariate analysis were used to describe data and determine significant predictors of behavioural risk factors of HIV infection.

**Results:**

Of 384 participants, 381 (99.2%) were sexually experienced, 353 (92.6%) were sexually active, and 168 (44.1%), reported condom use at last sex. The prevalence of ever-use of alcohol was 62%, and 68% of participants reported being current alcohol users. The prevalence of ever-use of marijuana was 15%, and 49% of participants reported being current marijuana users, with 12% reporting daily use. Age, marital status, working duration as a porter, transactional sex practices, and number of concurrent sexual partners were factors that were significantly associated with unprotected sex, alcohol consumption, and marijuana use (*P*<.05).

**Conclusions:**

Age, marital status, working duration, transactional sex practices, and number of concurrent sexual partners were significantly associated with unprotected sex, alcohol consumption, and marijuana use, among porters in this setting. The findings suggest the need for efforts to motivate sexually active male porters to engage in HIV prevention interventions, including condom use and reduction of multiple concurrent sexual partners, transactional sexual practices, alcohol consumption, and marijuana use.

## INTRODUCTION

HIV and AIDS remain a worldwide global health problem with an estimated 36 million people living with HIV globally.^1^ Sub-Saharan Africa is the region most affected by HIV/AIDS, contributing 75% of the global HIV burden.^1^ Preventing and controlling HIV/AIDS among youth aged 15–24 years is a current global challenge. According to the United Nations Population Fund (UNFPA), the youth population has surpassed 1 billion globally—the largest in human history.^2^ In Tanzania, almost two-thirds (65%) of the population is under the age of 24 years, and youth are affected most by HIV/AIDS with an overall prevalence of 7.9%.^3,4^

One of the major drivers of the high HIV prevalence among youth is early sexual activity. For example, a substantial proportion (85%) of unmarried youth in Tanzania are sexually experienced with low (12.2%) condom-use practices.^5^ Alcohol consumption and marijuana use are also becoming a major global public health problem, particularly among youth. According to the World Health Organization (WHO), alcohol consumption and marijuana use contribute 4% of the disability-adjusted life years of the global burden of disease.^6–9^ In sub-Saharan Africa, approximately 25% of young people are current drinkers and reported to consume 35 litres of pure alcohol annually.^10,11^ In Tanzania, the proportion of substance use, including alcohol consumption, is increasing, with 17.2% of urbanite youth current users.^10^ The high alcohol consumption among youth is compounded with risky sexual behaviours, such as multiple concurrent sexual partnerships and low or inconsistent condom use.^10^

In Kilimanjaro region, public health officers have identified 2 high-risk populations for HIV – female bar workers and male mountain-climbing porters. While several studies in this area have documented HIV risk behaviours among female bar workers^12,13^ and among youth in different settings,^5,16^ there is limited information pertaining to risk characteristics specifically of young male mountain-climbing porters, who may share many risk characteristics with other high-risk groups, such as long-distance truck drivers,^17,18^ fishermen,^19–21^ miners,^22,23^ and migrant farm workers.^24^

The estimated 17,000 porters of Mount Kilimanjaro are between the ages of 18 and 45 years, but are predominantly young men who are very mobile and face volatile income cycles.^14^ Evidence suggests that people who work in difficult environments, have seasonal jobs, and earn a higher-than-average living wage are at high risk of overspending money on alcohol and marijuana and are more likely to have multiple sexual partners. Such risky behaviours may lead to unsafe sex practices, posing a risk of HIV infection.^15^

The aim of this study was to assess the sociodemographic and other characteristics associated with behavioural risk factors of HIV infection among male mountain-climbing porters in Kilimanjaro region, Tanzania. The findings from this study will contribute to knowledge of risk characteristics of male porters of Mt. Kilimanjaro and may assist knowledge brokers, policy makers, and HIV prevention interventionists to develop effective strategies to influence behaviour change for prevention of HIV transmission among this high-risk group in this study setting.

## METHODS

### Study Population and Protocol

We conducted a cross-sectional study designed to assess sociodemographic and other characteristics associated with behavioural risk factors for HIV infection among male mountain-climbing porters in the Kilimanjaro region. According to a conversation with a representative from the Kilimanjaro Porters Assistance Project (February 2013), it is estimated that a total of 17,000 porters are registered with the 3 Kilimanjaro porters unions. A multistage sampling technique was used to obtain the estimated sample of participants. In the first stage, we randomly selected 7 of 14 tour companies. In the second stage, we obtained a list of all porters working in the 7 tour companies from the tour companies’ management and then used sampling proportionate to size technique to select a random sample of 384 participants. Sample size calculation was based on an HIV prevalence of 50%, as no other study has been done on behavioural risk factors for HIV among porters. Local interviewers were trained on obtaining informed consent, maintaining confidentiality, following interview procedures, and completing the questionnaire. Study participants, recruited between April and May 2013, completed in-person interviews in Kiswahili with the interviewers.

### Measures

Alcohol use was assessed by 11 questions whereby 4 questions were adapted from CAGE questionnaire.^25^ Marijuana use was assessed by 6 structured questions and a composite score was calculated. Participants self-reported sociodemo-graphic information included age, marital status, highest level of completed education, and income. Condom use was assessed by asking participants if they had had sex without a condom during the last sexual intercourse (1=yes; 2=no). A response of ‘yes’ indicated sexual risk behaviour. Transactional sex practices were assessed by asking participants if they had exchanged/paid/received a gift for sex during the last 6 months (1=no; 2=yes, received; 3=yes, given; 4=yes, received and given). Number of sexual partners was assessed by asking participants the number of casual sexual partners they had during the last 3 months. The number of any sexual partners was dichotomised and coded: ‘no multiple sexual partners’ (1 sexual partner) or ‘multiple sexual partners’ (>1 sexual partner).

### Statistical Analyses

Data were entered, cleaned, and analysed using Statistical Package for Social Science (SPSS) version 18 (SPSS, Inc., Chicago IL). Categorical data were summarised using frequency and percentages, while numerical data were summarised using measures of central tendencies with their respective measures of dispersions. Chi-square test was used to determine statistical associations between categorical variables. Bivariate logistic regression analysis was used to determine sociodemographic and other characteristics associated with behavioural risk factors of HIV infection among study participants. A P-value was considered statistically significant at the .05 level.

### Ethical Approval

The study and all study activities were approved by the Kilimanjaro Christian Medical University College Research Ethical Review Committee. Written informed consent was obtained. Participants were informed of their voluntariness to participate and freedom to withdraw at any point from the study.

## RESULTS

### Background Characteristics

#### Sociodemographic Characteristics

In total, 384 porters were enrolled in the study. The mean age of study participants was 31 years (standard deviation [SD], 6.6) (Table 1). More than half (n=199, 51.8%) of the study participants were married. Most (n=247, 64.3%) had primary education, and 60.2% earned an average monthly income of less than 400,000 Tanzanian Shillings (TZS) during high season (in 2012, US$1=1,600 TZS). More than half (n=219, 57.0%) of participants had been working as a porter for 3 to 5 years.

**TABLE 1.** Sociodemographic Characteristics of Respondents (N=384)

Variable	n (%)
Age (years)	
20-29	162 (42.2)
30-39	172 (44.8)
40-49	50 (13.0)
Level of education	
None	2 (0.5)
Primary	247 (64.3)
Secondary	126 (32.8)
Post-secondary	9 (2.3)
Marital status	
Married	199 (51.8)
Single	127 (33.1)
Cohabiting	54 (14.1)
Divorced	3 (0.8)
Widowed	1 (0.3)
Monthly income during high season (TZS) (n=383)	
<200,000	86 (22.4)
200,000-399,000	145 (37.8)
400,000-599,000	69 (18.0)
600,000-799,000	48 (12.5)
800,000+	36 (9.4)
Duration working as a porter (years)	
≤2	32 (8.3)
3-5	219 (57.0)
6-8	92 (24.0)
9-11	35 (9.1)
12 +	6 (1.6)
Duration of work, years, mean (standard deviation)	5.9 (4.6)

#### Sexual Characteristics

Nearly all participants (n=381, 99.2%) reported being sexually experienced (Table 2). Of the 381 respondents who reported being sexually experienced, 177 (46.5%) reported having sex 1 to 6 days prior to the interview and 353 (92.6%) said they had had sex during the last 3 months. More than half (54.6%) of sexually experienced respondents had had sex with their wives. The majority (85.8%) of participants did not report having sex in exchange for money or a gift during the last 6 months prior to the interview. Out of the 353 sexually active participants who had had sex during the last 3 months, 61 (17.3%) reported multiple concurrent sexual partners. Of the 381 sexually experienced participants, 168 (44.1%) reported condom use during their last sexual intercourse.

**TABLE 2.** Sexual Characteristics of Study Participants (N=384)

Variable (n)	n (%)
Ever had sex	
Yes	381 (99.2)
No	3 (0.8)
Last time had sex (381)	
1-6 days ago	177 (46.5)
7 days ago	65 (171.0)
1-11 months ago	101 (26.5)
≥12 months ago	38 (10.0)
Relationship to the woman with whom had last sex (381)	
Wife	208 (54.6)
Fiancée	35 (9.2)
Regular partner	77 (20.1)
Causal partner	54 (14.2)
Tourist	2 (0.5)
Commercial sex worker	5 (1.3)
Place you first met this woman (381)	
Own/friend's house	55 (14.4)
Family event	9 (2.4)
Bar/hotel	22 (5.8)
Park/company	11 (2.9)
Church	18 (4.7)
Other places	266 (69.8)
In last 6 months, exchanged/paid money/gift for sex (381)	
No	327 (85.8)
Yes, received	7 (1.8)
Yes, given	39 (10.2)
Yes, received and given	8 (2.1)
Relationship to the woman with whom paid/gave/received money/gift for sex (330)	
Tourist	5 (9.3)
Commercial sex worker	12 (22.2)
Bar/hotel worker	13 (24.1)
Sex partner/friend	24 (44.4)
Had multiple sexual partners in the past 3 months (353)	
Yes	61 (17.3)
No	292 (82.7)
Used condom at last sex (381)	
Yes	168 (44.1)
No	215 (55.9)

### Prevalence of Alcohol and Marijuana Use

Of the total 384 participants, 237 (61.7%) reported ever drinking alcohol (Table 3). Of these, 161 (67.9%) reported being current drinkers. Of the 161 current drinkers, 81 (50.3%) reported drinking 2 to 7 times per week and drinking on average 4 bottles (SD, 3; range, 1–25) during a typical day. The majority (n=136, 84.5%) of current drinkers reported drinking beer, and 6 (3.7%) took 6 or more drinks at 1 occasion daily.

**TABLE 3.** Prevalence of Alcohol Use Among Study Participants (N=384)

Variable (n)	n (%)
Ever drank alcohol (384)	
Yes	237 (61.7)
No	147 (38.3)
Currently drinking alcohol (237)	
Yes	161 (67.9)
No	76 (32.1)
Frequency of drinking (161)	
Monthly or less	15 (9.3)
2-4 times a month	65 (40.4)
2-3 times a week	57 (35.4)
4+ times a week	24 (14.9)
Type of alcohol (161)	
Beer	136 (84.5)
Liquor	3 (1.9)
*Mbege* (local brew)	21 (13.0)
Both	1 (0.6)
Frequency of having 6 or more drinks on 1 occasion (161)	
Never	106 (65.8)
Once per month	25 (15.5)
2-3 times per month	15 (9.3)
Weekly	9 (5.9)
Daily/almost daily	6 (3.7)
Number of drinks taken on a typical day, mean (standard deviation) (161)	4.12 (3.14)

Of the 384 participants, 57 (14.8%) reported to have ever used marijuana (Table 4). Mean age at first time using marijuana was 18 years (SD, 5.4). The youngest age reported to have started using marijuana was 7 years while the oldest age was 45 years. The prevalence of reported current marijuana use was 49.1% (n=28). Out of 28 respondents who reported current marijuana use, 12 (42.9%) reported daily use.

**TABLE 4.** Prevalence of Marijuana Use Among Study Participants (N=384)

Variable (n)	n (%)
Ever used marijuana (384)	
Yes	57 (14.8)
No	327 (85.2)
Currently using marijuana (57)	
Yes	28 (49.1)
No	29 (50.9)
Age first started using marijuana (57)	
≤10	3 (5.3)
11-20	42 (73.7)
21-30	11 (19.3)
31-40	0 (0.0)
41 +	1 (1.8)
Usual frequency of using marijuana (28)	
Every day	12 (42.9)
2-3 times a week	9 (32.1)
2-3 times a month	5 (17.9)
Once in a month	2 (7.1)

**TABLE 5.** Sociodemographic Characteristics Associated With Behavioural Risk Factors for HIV Among Study Participants (N=384)

	Behavioural Risk Factors				
Characteristics	Total (n)	Did Not Use Condom During Last Sex OR (95% CI)	Had Multiple Concurrent Sexual Partner OR (95% CI)	Use Alcohol OR (95% CI)	Use Marijuana OR (95% CI)
Age (years)					
20-29	162	1	1	1	1
30-39	172	4.60 (2.76, 7.73)	0.39 (0.21, 0.73)	1.71 (1.12, 2.72)	0.54 (0.30, 0.99)
40-49	50	20.20 (4.75, 86.10)	0.61 (4.75, 86.10)	1.63 (0.84, 3.16)	0.25 (0.07, 0.85)
Marital status					
Married	199	1	1	1	1
Single	127	0.06 (0.03, 0.11)	3.27 (1.76, 6.07)	1.01 (0.64, 1.50)	5.76 (2.78, 11.92)
Cohabiting	54	0.47 (0.20, 1.12)	1.67 (0.72, 3.89)	1.32 (0.70, 2.48)	5.42 (2.27, 12.95)
Divorced/widowed	4	0.19 (0.02, 2.17)	4.00 (0.35, 45.94)	NC	8.55 (0.72, 10.66)
Monthly income during high season (TZS)					
<200,000	86	1	1	1	1
200,000-399,000	145	1.60 (0.65, 3.90)	0.68 (0.22, 2.12)	1.62 (0.69, 3.78)	1.55 (0.42, 5.71)
400,000-599,000	69	1.36 (0.58, 3.16)	0.79 (0.27, 2.29)	0.95 (0.86, 4.40)	1.60 (0.45, 5.67)
600,000-799,000	48	2.52 (0.81, 7.84)	1.04 (0.29, 3.75)	1.99 (0.72, 5.47)	0.71 (0.13, 3.80)
800,000+	36	3.43 (1.07, 11.01)	1.16 (0.34, 3.97)	1.68 (0.63, 4.49)	1.11 (0.24, 5.09)
Duration working as a porter (years)					
≤2	32	1	1	1	
3-5	219	2.61 (1.48, 4.60)	1.19 (0.54, 2.59)	1.83 (1.07, 3.14)	0.54 (0.26, 1.15)
6-8	92	3.68 (1.76, 7.70)	1.31 (0.54, 3.18)	4.14 (2.03, 8.44)	0.92 (0.40, 2.10)
9-11	35	4.24 (1.77, 10.13)	1.71 (0.67, 4.36)	1.36 (0.67, 2.76)	0.63 (0.23, 1.72)
12+	6	4.03 (1.65, 9.83)	0.33 (0.07, 1.58)	4.03 (1.65, 9.83)	
In last 6 months, exchanged/paid money/gift for sex					
No	327	1	1	1	1
Yes, received	7	1.49 (1.11, 2.25)	3.35 (1.62, 17.97)	0.49 (0.11, 2.25)	2.79 (1.52, 14.85)
Yes, given	39	1.91 (0.90, 4.06)	11.52 (5.49, 24.16)	1.91 (0.90, 4.06)	3.10 (1.46, 6.59)
Yes, received and given	8	1.10 (0.26, 4.68)	13.96 (3.19, 61.17)	1.10 (0.26, 4.68)	0.99 (0.12, 8.31)
Number of any sexual partner in the past 3 months					
≤1	292	1	-	1	1
More than one	61	1.38 (1.21, 0.67)	-	1.51 (0.83, 2.74)	1.99 (1.00, 3.96)
Total		73.0% (n=278)	17.3% (n=61)	61.7% (n=237)	14.8% (n=57)

Abbreviations: CI, confidence interval; NC, not calculated; OR, odds ratio.

### Sociodemographic and Other Characteristics Associated With Behavioural Risk Factors of HIV Infection Among Study Participants

The 4 behavioural risk factors of HIV infection in this study were condom use during the last sexual intercourse, having multiple concurrent sexual partners, and use of alcohol and marijuana. Age was significantly associated with all 4 of these behavioural risk factors (*P*<.05): older participants were more likely than younger participants (20–29) to have not used condoms at last sex (30–39 year age group: odds ratio [OR] 4.60; 95% confidence interval [CI], 2.76–7.73; 40–49 year age group: OR 20.20; 95% CI, 4.75–86.10) but less likely to have multiple concurrent sexual partners (OR 0.39; 95% CI, 0.21–0.73 vs. OR 0.61; 95% CI, 4.75–86.10, respectively). Similarly, older participants were more likely than younger participants to consume alcohol (OR 1.71; 95% CI, 1.12–2.72 vs. OR 1.63; 95% CI, 0.84–3.16, respectively) and to use marijuana (OR 0.54; 95% CI, 0.30–0.99 vs. OR 0.25; 95% CI, 0.07–0.85, respectively).

Marital status was significantly associated with condom use during last sexual intercourse, having multiple concurrent sexual partners, and marijuana use (*P*<.05). Compared with married participants, those who were single were less likely to have had unprotected sex during last sexual intercourse (OR 0.06; 95% CI, 0.03–0.11), but were more likely to have multiple concurrent sexual partners (OR 3.27; 95% CI, 1.76–6.07) and to use marijuana (OR 5.76; 95% CI, 2.78–11.92).

In general, the longer respondents worked as a porter, the higher the likelihood of them not using condoms as last sex (*P*<.001).

Receiving money or gifts for sex during the last 6 months was significantly associated with having unprotected sex during the last sexual intercourse (OR 1.49; 95% CI, 1.11–2.25). In addition, receiving or giving money or gifts for sex was significantly associated with having multiple concurrent sexual partners and with marijuana use (*P*<.05).

Furthermore, the number of any sexual partners in the past 3 months was significantly associated with unprotected sex during the last sexual intercourse (*P*=.001). Respondents with more than 1 sexual partner were more likely not to use condoms in their last sexual intercourse (OR 1.38; 95% CI, 0.21–0.67).

Level of income was not significantly associated with any of the 4 studied behavioural risk factors (*P*>.05).

## DISCUSSION

The study findings have shown that certain sociodemo-graphic characteristics, as well as paying or receiving money or gifts for sex, are associated with behavioural risk factors for HIV infection among porters in Kilimanjaro region, Tanzania. Overall, the majority of porters were sexually experienced, more than half (56%) reported they did not use a condom during their last sexual intercourse, and almost one-fifth (17%) reported having multiple concurrent sexual partners.

Overall, older respondents were more likely to have not used condoms, so unprotected sex increased with increasing age, but the likelihood of having multiple concurrent sexual partnerships decreased with increasing age. The most probable explanation to these observations may be due to the fact that young male porters tend to use condoms because they also engage in multiple concurrent sexual partnerships, hence perceive themselves being at high risk of HIV infection.^5^,^26^,^27^ In contrast, older male porters are less likely than older porters to use condoms because they may be in a regular sexual partnership. Additionally, this observation of high condom use among younger participants can possibly be due to exposure to sensitisation messages on condom use.^4^ This finding is in contrast with findings from previous investigators on youth's sexual activity in Tanzania, who found that condom use was higher among older respondents.^4^,^16^

Marital status was associated with unprotected sex, multiple concurrent sexual partnerships, and marijuana use. The likelihood of condom use was higher among unmarried participants than their married counterparts. Furthermore, unmarried participants were more likely to have multiple concurrent sexual partners and to smoke marijuana. This finding is consistent with behavioural studies assessing motivating factors associated with condom use among youth, which found that respondents who perceived higher susceptibility to HIV infection through their risky behaviours were more likely to report condom use.^5^,^16^

Transactional sex was associated with unprotected sex, having multiple concurrent partners, and marijuana use. Porters who reported to have paid and/or received money or a gift in exchange for sex were more likely to report unprotected sex and to smoke marijuana. The finding that transactional sexual practice was associated with unprotected sex is consistent with several studies among youth in different settings. Existing evidence on HIV infection in sub-Saharan Africa shows that transactional and transgender sexual practices are 2 major drivers for the high HIV prevalence in the general population, including youth.^1^ Additionally, the observed risk behaviour of mixing unprotected sexual practices and marijuana smoking underscores the importance of skills development for safer sex among porters in this setting. An intervention designed to increase motivation for condom use while addressing the mixed-risk behaviours may be an effective approach.^1^, ^28^,^29^

Finally, the number of concurrent sexual partners in the last 3 months was associated with non-condom use among porters. Porters who reported more than 1 concurrent sexual partner during their last sexual intercourse were more likely to engage in unprotected sex. This observation is consistent with studies on condom use among youth,^1,30,31^ and raises concern that porters in this setting are at high risk of contracting HIV. Indeed, low rates of condom use coupled with high rates of multiple concurrent sexual partners may increase the spread of HIV among male porters.

### Limitations

Although this study addressed a number of behavioural risk factors associated with HIV infection among Kilimanjaro mountain porters, some risk factors were not explored. This included knowledge of HIV transmission and prevention, sexual practices, HIV status, and history of other sexually transmitted diseases. Further studies addressing these risk factors are important to better understand this at-risk population for HIV.

This study has other certain limitations. First, the cross-sectional study design that we used is not adequate for measuring the directionality of associations found and, therefore, cannot account for potential confounders. Second, we asked respondents to recall events that had occurred in the distant past. Recall bias may be a limitation, particularly among older porters who may be unable to remember the exact timing of their early sexual activities. Third, the study relied on self-reports. It is known that the self-reported behaviour is subject to reporting bias, which may overestimate or underestimate the effects of association and the validity of the findings. In particular, due to marijuana use being illegal in Tanzania, it was likely under-reported. Finally, given that this study was conducted in a particular location and among male porters, it may not be applicable to other settings or the general population. Nevertheless, the study findings provide an important insight into male mountain climbing porters’ behavioural risk factors for HIV infection.
